# The Preparation of Superhydrophobic Polylactic Acid Membrane with Adjustable Pore Size by Freeze Solidification Phase Separation Method for Oil–Water Separation

**DOI:** 10.3390/molecules28145590

**Published:** 2023-07-22

**Authors:** Yan Zhang, Tianyi Sun, Dashuai Zhang, Shishu Sun, Jinrui Liu, Bangsen Li, Zaifeng Shi

**Affiliations:** 1 Key Laboratory of Water Pollution Treatment & Resource Reuse, Hainan Normal University, Haikou 571158, China; zy936457505@163.com (Y.Z.); sss_1996@163.com (S.S.); jinruiliu1996@163.com (J.L.); bangsenli@163.com (B.L.); 2 College of Chemistry and Chemical Engineering, Hainan Normal University, Haikou 571158, China

**Keywords:** PLA membrane, freeze solidification phase separation method, adjustable pore size, superhydrophobic, oil–water separation

## Abstract

An environmentally friendly pore size-controlled, superhydrophobic polylactic acid (PLA) membrane was successfully prepared by a simpler freeze solidification phase separation method (FSPS) and solution impregnation, which has application prospects in the field of oil–water separation. The pore size and structure of the membrane were adjusted by different solvent ratios and solution impregnation ratios. The PLA-FSPS membrane after solution impregnation (S-PLA-FSPS) had the characteristics of uniform pore size, superhydrophobicity and super lipophilicity, its surface roughness Ra was 338 nm, and the contact angle to water was 151°. The S-PLA-FSPS membrane was used for the oil–water separation. The membrane oil flux reached 16,084 L·m^−2^·h^−1^, and the water separation efficiency was 99.7%, which was much higher than that of other oil–water separation materials. In addition, the S-PLA-FSPS membrane could also be applied for the adsorption and removal of oil slicks and underwater heavy oil. The S-PLA-FSPS membrane has great application potential in the field of oil–water separation.

## 1. Introduction

With the rapid development of economy and society, industrial wastewater, domestic wastewater discharge, and oil leakage have caused serious damage to the environment and ecology, and affected the life of aquatic organisms and human health [1,2]. To solve the problem of oil pollution, oil–water separation has become an important research topic [3,4,5]. Membrane separation technology with the advantage of simple operation is one of the most effective methods for oil–water separation [6,7]. However, the traditional membranes with small pore sizes, poor separation rate, and poor separation efficiency, limit the scope of the applications. Therefore, there is an urgent need to develop new types of selectively separating various oil–water mixtures. The key to oil–water separation material is to design and synthesize materials with special wettability [8,9,10]. In recent years, people have designed various superhydrophobic and superoleophilic [11], superhydrophilic and underwater superoleophobic [12], and superhydrophobic and superoleophobic [13,14] materials, such as polymer membranes [15] and ceramic membranes [16]. Typically, polymer membrane materials with hydrophobic properties are built with a rough micro-structure [17,18,19] or decreased surface energy by surface modification [20,21,22]. However, these materials are usually non-degradable, fluorinated, or non-recyclable, and prone to secondary environmental pollution after use. Therefore, it is of great significance to prepare environmentally friendly polymer materials for the separation of oil–water.

At present, the preparation methods of separation of oil–water membranes include electrospinning [23,24], phase separation [25,26], layer-by-layer assembly [27], etc. By contrast, the membranes prepared by the phase separation method has the advantages of simplicity. Wang et al. [28]. successfully induced micropatterns with micro- or nanometer-scale concavities on the polymer film surface using a facile phase separation method. This further indicates that it is feasible to prepare the porous polymer membrane by the phase separation. In addition, the improvement of hydrophobicity usually requires the addition of nano-materials and fluorine-containing components [29], which have some problems such as high cost, complex operation, and secondary environmental pollution. Compared with these modification methods, the solution impregnation induction is one of the simplest and efficient methods to improve the superhydrophobicity of a membrane.

Polylactic acid (PLA), also known as polylactide, is a polyester polymerized from lactic acid [30]. Lactic acid is derived from the fermentation of starch (such as corn and rice), and can also be obtained from cellulose, kitchen waste, or fish waste as raw materials [31]. PLA has a wide range of raw material sources and excellent biodegradability. Products made of PLA can be directly composted after use, and are eventually completely degraded into CO_2_ and H_2_O, meeting the requirements of sustainable development [32,33]. Most PLA membrane separation materials are prepared by freeze-vacuum drying [34,35], but there are problems with energy consumption and industrial production. In this study, a new freeze solidification phase separation method was proposed, which only formed the membrane by freezing and curing at room temperature. The simple preparation method is more competitive.

To develop environmentally friendly high-efficiency and stable polymer materials for oil–water separation, a new type of freeze solidification phase separation method was used to prepare superhydrophobic porous S-PLA-FSPS membrane. Induced freezing solvent was used as pore-forming agent. The homogeneous mixed solution of PLA and 1,4-dioxane (DiOX) was placed in low temperature (below the freezing point of DiOX). During the DiOX freezing crystallization, the DiOX between the PLA polymer chains induced PLA to form the three-dimensional polymer skeleton. Then, the phase separation occurred at room temperature, and the DiOX solvent was evaporated to obtain the PLA membrane. By this method, the PLA-FSPS membranes had uniform pore size, high porosity, super-oleophilic, and hydrophobic properties. Moreover, the effects of the different solvent ratios and solution impregnation on the regulation of pore size and superhydrophobicity were explored. The S-PLA-FSPS membrane had an efficient and stabile oil–water separation effect, and the mechanism of oil–water separation was analyzed, providing the possibility for practical wastewater treatment.

## 2. Results and Discussion

### 2.1. Optimization of Preparation Method

**Figure 1:**
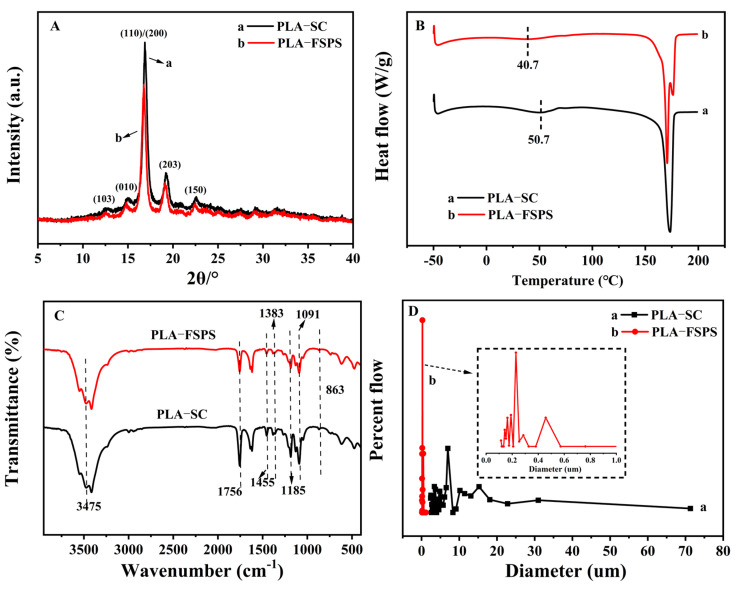
XRD (**A**), DSC curve (**B**), FT-IR spectrum (**C**) and pore size test (**D**) of PLA-SC and PLA-FSPS.

The PLA membranes were prepared by the solution casting method (PLA-SC) and freeze solidification phase separation method (PLA-FSPS), respectively. The crystal structure and crystallization behavior of PLA-FSPS and PLA-SC were characterized by XRD and DSC curves, respectively. As shown in Figure 1A, the typical α-PLA crystal diffraction peaks corresponded to (110)/(200) and (203) at 2θ values around 16.8° and 19.2°, respectively. The diffraction peaks of PLA prepared by PLA-FSPS shifted to a lower angle, indicating the increase of interplanar spacing and the stacking between molecular chains. The weaker diffraction peaks at 12.4°, 14.8°, and 22.3° corresponded to the (103), (010), and (150) of α′-PLA crystal form [36], respectively. Therefore, the obtained PLA was composed of α′ and α mixed crystal types. By comparing the two different preparation methods, it could be found that the diffraction peaks of PLA-FSPS was the half-peak width reduced. This was because the freezing process could promote crystallization under the action of internal stress, and the crystal structure became more regular and perfect [37].

The DSC heating test was carried out on PLA prepared by two different preparation methods. Figure 1B shows that the glass transition temperature (T_g_) of the PLA-SC sample was about 50.7 °C, and a melting single peak was observed with the melting temperature (T_m_) of 173.5 °C. T_g_ of the PLA-FSPS sample was also about 40.7 °C, while the double melting peaks appeared at 170.6 °C. The appearance of the double peak phenomenon might be due to the rearrangement of the imperfect crystal molecular chain during the crystallization process, and the formation of a perfect crystal during the melting process [38,39].

Seen from the FT-IR spectrum (Figure 1C), the C=O stretching vibration adsorption peak of PLA appeared at 1756 cm^−1^. The absorption peaks of symmetric stretching vibration and asymmetric stretching vibration of C-O appeared at 1091 cm^−1^ and 1185 cm^−1^, respectively. The infrared absorption peak at 1455 cm^−1^ was assigned to the bending vibration peak of –C–H–. The C–C vibrational absorption peak in the molecular chain appeared at 863 cm^−1^. The characteristic peak of –CH_3_ and –OH appeared at 1383 cm^−1^ and 3475 cm^−1^, respectively. Thus, the different preparation methods have no effect on the change of molecular structure.

The pore size and pore size distribution of PLA samples were determined by the capillary flow pore size analyzer. As shown in Figure 1D and Table S1 (Supporting Information), PLA-SC had a wide pore size distribution range with the average pore size of 25.86 μm, the maximum and minimum pore size were 71.30 μm and 2.35 μm. The average pore size of the PLA-FSPS membrane was 0.46 μm, and the maximum and minimum pore size were 0.54 μm and 0.25 μm. In comparison, the PLA-FSPS was smaller pore size and uniform distribution. The porosity of PLA-FSPS was 77.60%, which was 26.59% higher than that of PLA-SC (Table S1). Therefore, the PLA prepared by the freeze solidification phase separation method had the uniform pore size distribution and high porosity. Moreover, due to the formation of more voids, the PLA-FSPS had higher reflectance than that of the PLA-SC (Figure S1), which was consistent with the results of the porosity test. 

**Figure 2:**
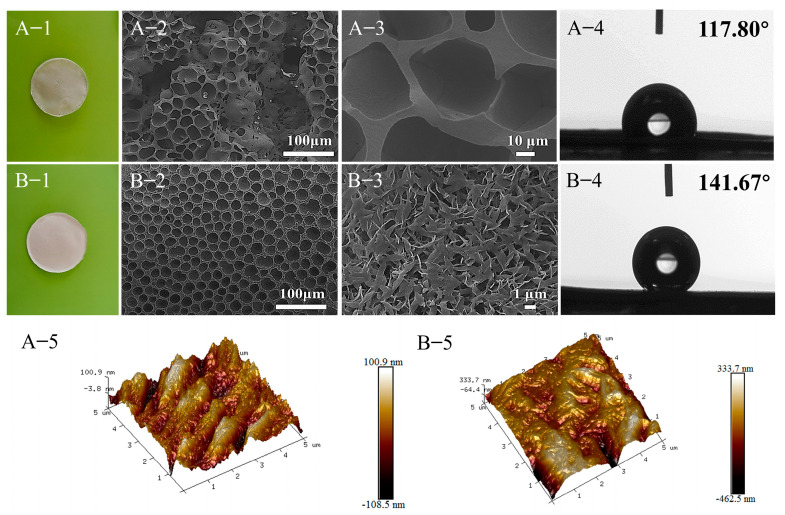
Digital photographs (**A-1**,**B-1**), SEM (**A-2**,**A-3**,**B-2**,**B-3**), CA (**A-4**,**B-4**), and AFM images (**A-5**,**B-5**) of PLA-SC (**A**) and PLA-FSPS (**B**).

**Figure 3:**
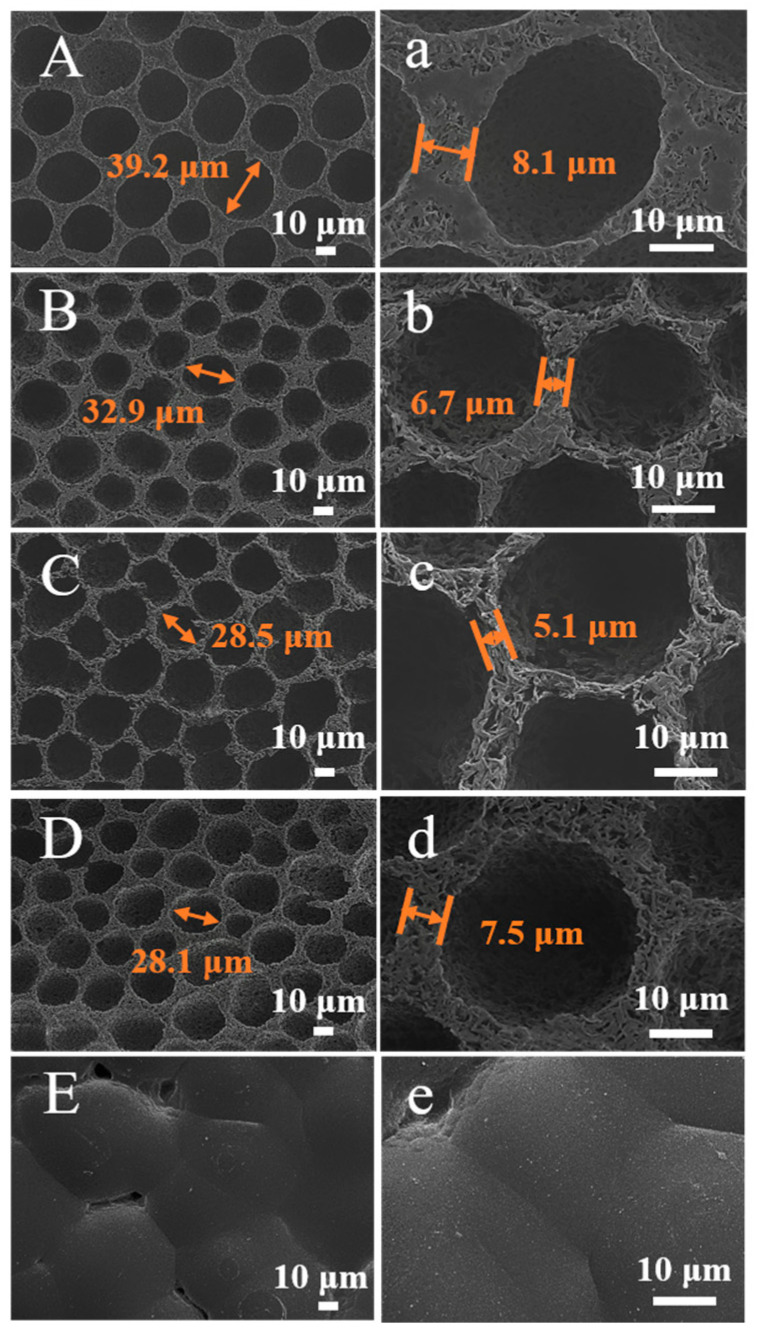
SEM test images (**A**–**E**) and magnification (**a**–**e**) of PLA-FSPS membranes with different solvent ratios.

**Figure 4:**
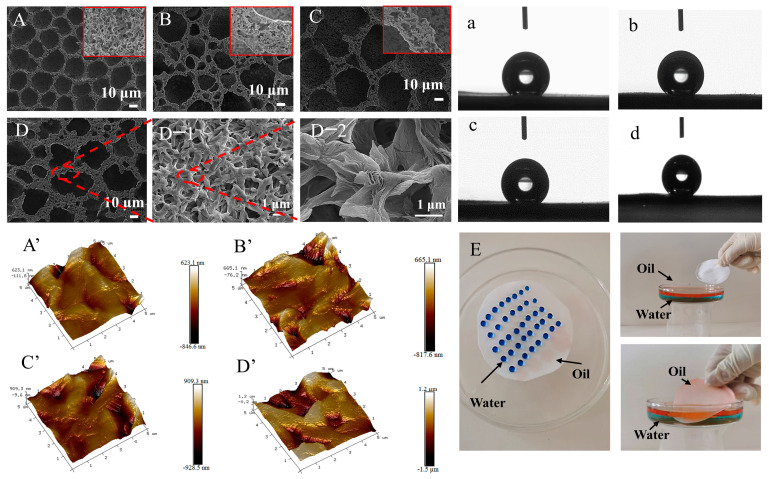
No solution impregnation (**A**,**a**,**A’**), water (**B**,**b**,**B’**), acetic acid (**C**,**c**,**C’**), deionized water and acetic acid mixture (**D**,**d**,**D’,D-1,D-2**) induced SEM, CA, AFM images of the membrane, digital photograph of membrane wettability observation (**E**).

**Figure 5:**
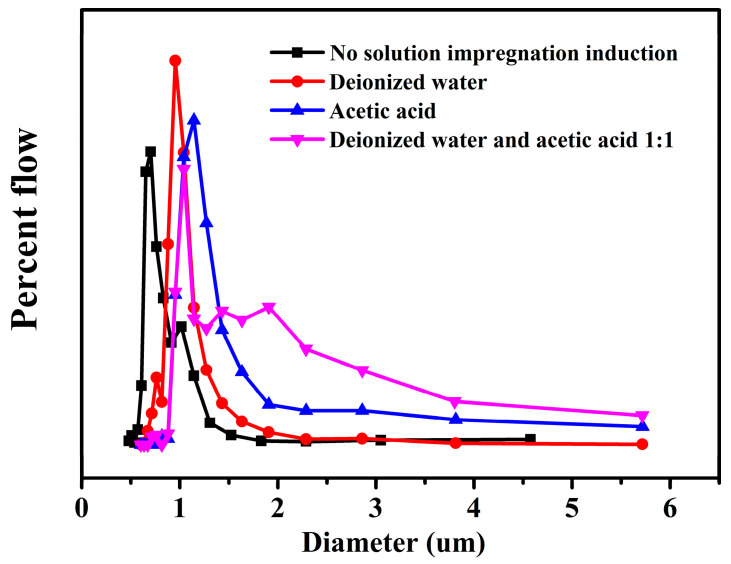
Pore size distribution of solution impregnation induced membranes.

The effects of different preparation methods on the surface morphology and roughness of membranes were analyzed by the photographs, SEM, AFM, and CA and shown in Figure 2. Compared with the solution casting method (Figure 2(A-1)), the PLA-FSPS (Figure 2(B-1)) was more flat, smooth, and whiter. The surface of the PLA-SC was closely packed with crystal balls [40], which had more macroporous structures and uneven distribution (Figure 2(A-2,A-3)). In addition, as seen from Figure 2(A-4,A-5), the roughness (Ra) and contact angle were 23.4 nm and 117.80° ± 4.78°, respectively. As for the PLA-FSPS, the pores formed on the surface were multi-micro or nano-scale size and more uniform (Figure 2(B-2,B-3)), the roughness (Ra) was 78.4 nm (Figure 2(B-5)), and the contact angle was 141.67° ± 1.45° (Figure 2(B-4)). Through the comparison of different preparation methods, it is found that the PLA-FSPS has more dense and uniform pores. The surface morphology of the porous structure led to the increase of roughness and contact angle, and the improvement of hydrophobicity. Combined with the surface and cross section of the PLA-FSPS membrane (Figure S2), the formation possibility of the porous membrane was analyzed. Induced freezing solvent was used as pore-forming agent. When the solvent reached freezing point solidification at low temperature, PLA polymer chains were fixed by the solvent and crystallized to form a three-dimensional polymer skeleton. At room temperature, the DiOX solvent was evaporated to obtain the porous PLA membrane. Compared with the reported preparation of porous PLA materials by the freeze–vacuum drying method [34,35,41], the freeze solidification phase separation method omitted the harsh process of vacuum drying, which was more beneficial to energy saving and industrial production. In practical applications, the thermal stability and mechanical properties of membranes played an important role. The TG and DTG curve of the PLA-FSPS membrane under nitrogen atmosphere (Figure S3), PLA-FSPS have only one weightlessness stage due to thermal degradation. The initial and end final degradation temperatures of PLA membranes were 328 °C and 381 °C, respectively. The maximum stress and strain of the membrane were 4.04 MPa and 2.1% (Figure S4), respectively, providing reference for practical application.

The PLA-FSPS with uniform porosity, high porosity, and hydrophobicity was obtained, which could be used as an oil–water separation material. The oil–water separation test of PLA-SC and PLA-FSPS were investigated and shown in Figure S5. The membrane flux of PLA-SC could not achieve oil–water separation due to excessive pore size and poor hydrophobicity. Remarkably, the PLA-FSPS had excellent oil–water separation property with a membrane flux of 2923 L·m^−2^·h^−1^ and a water separation efficiency of 96.4%. To sum up, the freeze solidification phase separation method was optimized to prepare PLA-FSPS membrane in the work below. In addition, to further improve the membrane flux and separation effect, the membrane pore size and surface wettability was regulated in the next step.

### 2.2. Control of the Pore and Surface Structure

#### 2.2.1. Effect of Different Solvent Ratios

Based on the above analysis, the multi-level micro-nanoporous PLA-FSPS membranes prepared by the simple freeze solidification phase separation method could be applied to the oil–water separation field. Membrane aperture has an important effect on membrane flux. In order to obtain PLA-FSPS membrane with excellent flux performance, DCM additive was introduced to control the pore size of PLA-FSPS membrane by adjusting the ratio of DiOX and DCM. The volume ratios of DiOX and DCM (10:0, 9:1, 4:1, 1:1, and 0:10) were adjusted during the preparation process. As shown in Figure 3A–E,a–e, the change of the solvent ratio resulted in the change of the surface morphology of the PLA membranes. With the introduction of DiOX in the solvent, a large number of pores appeared on the membrane surface, and the size of the surface pores ranged from 20 to 40 μm. When only DCM was used as the solvent, the membrane was dense and had no pores. When the volume ratio of DiOX and DCM was 4:1, the distance between holes became much smaller, and the membrane pores were much denser. To understand the effect of DiOX and DCM solvents on membrane surface wettability, the contact angles of these membranes were tested by CA (Figure S6A’–E’). With the increase of DCM solvent, the contact angle decreased from 141.67° to 90.08°. This was mainly due to the change in wettability and the size and spacing of pores on the surface caused by the difference in solvent ratios.

To understand the effect of DiOX and DCM solvents on pore size and pore size distribution (Table 1), the pore size and pore size distribution of different PLA samples were determined. The pore size distributions of PLA with the DiOX:DCM volume ratios of 10:0, 9:1, 4:1, and 1:1 were mainly concentrated in 0.1~0.6 μm, 0.9~2 μm, 0.5~2.1 μm, and 0.1~0.5 μm, respectively (Figure S7), and the average pore diameters of which were 0.46 μm, 0.98 μm, 1.32 μm, and 0.35 μm, respectively. Therefore, the regulation of pore size, pore size distribution, and morphology could be achieved by adjusting the ratios of solvent. Among them, when the ratio was 4:1, the pore size was the largest and the pore size distribution was the widest. When the solvent was only DCM, the membrane became dense, and its pore size could not be measured by this instrument. The reason for the formation of non-porous membrane was that the freezing temperature did not reach the freezing point (−95 °C) of DCM solvent, and the non-frozen DCM solvent could not fix the distance between PLA molecular chains, so the closely packed polymer membrane was obtained after the solvent volatilization. Through the oil–water separation experiment, PLA-FSPS (4:1) with abundant pores have good membrane flux (Figure S8). Thus, in the subsequent experiment, the PLA-FSPS membrane was prepared by the freeze solidification phase separation method with the volume ratio of DiOX to DCM at 4:1.

#### 2.2.2. Effect of the Impregnating Solution

In order to further improve the superhydrophobicity of PLA, the S-PLA-FSPS was obtained by solution impregnating the semi-cured PLA-FSPS. The deionized water, acetic acid, and a mixture of deionized water and acetic acid with a volume ratio of 1:1 was selected as the solution impregnation, respectively, and the induction effects of the solution impregnation were analyzed. As shown in the SEM images (Figure 4A–D), by deionized water (Figure 4B) and mixed solution (Figure 4D) inducing, the pores on the membrane surface were of different sizes and irregular shapes. After the acetic acid treatment, the pores on the membrane surface enlarged, and changed from regular circles to ellipses (Figure 4C). According to the large scanning magnification (Figure 4(D,D-1,D-2)), the solution induced by solution impregnation showed a star-like wrinkled structure connected by lamellar structures. Visibly, the solution impregnation had a great influence on the surface morphology of the PLA membrane. In the solution impregnation process, due to the different composition and concentration of the polymer solution and impregnation solution, the solvent and impregnation solution in the semi-cured PLA membrane began to diffuse bidirectionally under the action of the concentration difference [42,43]. The morphology of the semi-cured PLA was changed, and the nano-scale wrinkled structures and a large number of pore structures appeared on the membrane surface in the above process (Figure 4(D-1,D-2)). Thus, it could be expected that the surface micro-roughness and contact angle increased after solution impregnation.

To verify the above analysis, the AFM surface roughness test was also performed on these membranes (Figure 4A’–D’). The membranes’ roughness Ra of without induced solution impregnation, water, acetic acid and mixed solution induced membranes were 78.4 nm, 177 nm, 171 nm, and 338 nm, respectively. Compared with no solution impregnation induced roughness, the roughness of the membrane induced by the mixed solution increased by 259.6 nm. Apparently, the roughness of the membrane surface greatly increased after the solution impregnation induced, particularly the membrane induced by the mixture of deionized water and acetic acid. Presumably, the semi-cured PLA membrane was impregnated in a mixed solution, the mixed solution diffused between the polymer and solvent. The acetic acid induced the semi-cured PLA molecular chains to entangle with each other, and water molecules accelerated the phase separation rate. Thus, more micro–nano structures with star-shaped folds were formed on the surface, which greatly increased the roughness.

In addition, these membranes also had good superhydrophobicity (Figure 4a–d). The contact angles of the membranes induced by no solution impregnation, deionized water, acetic acid, and mixed solution were 140.67°, 145.73°, 145.07°, and 151.00°, respectively. The contact angle is closely related to the surface roughness of materials [44,45]. The increase of contact angle was mainly because the solution impregnation promoted the change of surface morphology and the increase of membrane roughness. The membrane induced by the mixed solution had the best superhydrophobic property, which was consistent with the test results of SEM and AFM. Significantly, the contact angle of the membrane induced by the mixed solution to oil was 0°, indicating that it also had good superoleophilicity (Figure S9). Additionally, the water was stained with methylene blue dye, and the oil was stained with Sudan red. The wettability of the mixture induced membrane was observed and shown in Figure 4E. The digital photos showed the membrane could only be infiltrated by the red oil, which further confirmed its good superhydrophobicity and superoleophilicity (Video S1).

For the oil–water separation, the membrane flux is an important measure. Therefore, the pore size and pore size distribution of the membrane is of great significance. It can be seen from Figure 5 and Table S2 that the pore size distribution of the membrane induced by solution impregnation was shifted to the large pore size, and the maximum pore size was about 11 μm. The membrane induced by the mixed liquid had the largest average pore size of 4.05 μm, which was beneficial to membrane flux.

### 2.3. Oil–Water Separation Performance

**Figure 6:**
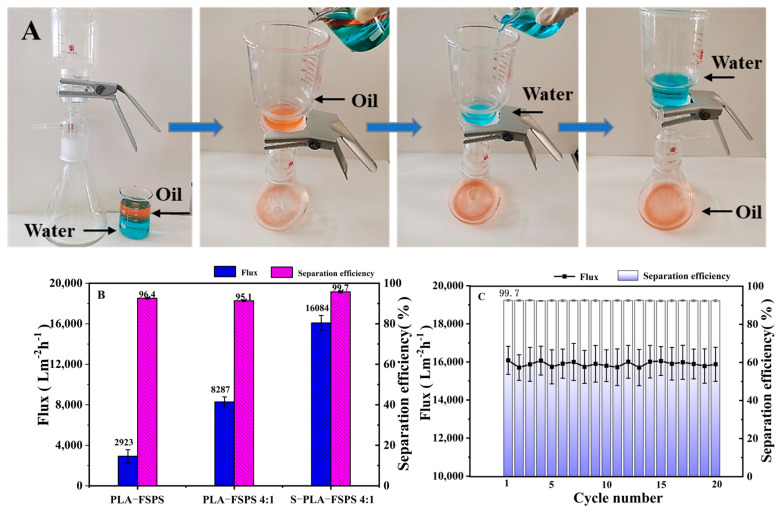
Oil–water separation device and process (**A**), PLA-FSPS, PLA-FSPS 4:1, and S-PLA-FSPS 4:1 membrane flux and separation efficiency (**B**), S-PLA-FSPS cycle test (**C**).

The PLA membranes with the good superhydrophobicity, superoleophilicity, and suitable pore structure have potential application prospects in oil–water separation [46]. The oil–water separation property of the PLA membrane was investigated by using an oil–water separator under gravity driving force, and the operation flow of oil–water separation is shown in Figure 6A. The prepared PLA membrane was placed in a separator, and the mixture of oil–water with the volume ratio of 1:1 was successfully separated. In addition, the membrane flux and oil–water separation efficiency of PLA-FSPS, PLA-FSPS (4:1), and S-PLA-FSPS are present in Figure 6B. After adjusting the proportion of solvent and impregnating the solution, both the membrane flux and oil–water separation efficiency were greatly improved. The membrane flux of S-PLA-FSPS was 16,084 L·m^−2^·h^−1^, and the water separation efficiency further promoted to 99.7%. Thus, this was further confirmed that the solution impregnation induction played an important role in improving the membrane flux. Moreover, the S-PLA-FSPS membrane still exhibited the excellent membrane flux and high separation efficiency after 20 cycles (Figure 6C), making it a strong practical application potential in the field of oil–water separation. Compared with the membrane flux values of the oil–water separation materials prepared in recent studies (as shown in Table 2), the S-PLA-FSPS membrane prepared in this study had significant advantages.

To sum up, the S-PLA-FSPS membrane had a good oil–water separation effect, which could be explained as follows. The oil–water separation performance of membranes was determined by three basic elements: wetting properties, membrane pore size, and breakthrough pressure. The breakthrough pressure refers to the maximum pressure required for the liquid penetrating into the membrane pores, and it can be expressed as the Young–Laplace equation [54]:

ΔP = −(2 γ_L_cosθ/r_p_) (1)

where, ∆P is the breakthrough pressure, γ_L_ is the surface tension of the liquid, θ is the contact angle of the liquid, and r is the pore radius.

**Figure 7:**
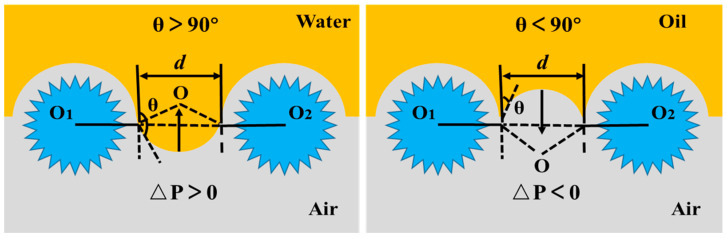
Water and oil intrusion pressures in “oil removal” mode.

It can be seen from Equation (1) that the surface wettability of the membrane had a very important influence on the breakthrough pressure. The oil–water separation principle is shown in Figure 7 [54,55]. The S-PLA-FSPS membrane was superhydrophobic, the water contact angle was greater than 90°, and the corresponding ΔP was greater than 0. Thus, the S-PLA-FSPS membrane could withstand a certain pressure before being wetted by water, and the water can be retained and cannot penetrate into the membrane pores. Meanwhile, the S-PLA-FSPS also had super-oleophilic property, the oil contact angle was less than 90°, and the corresponding ΔP was less than zero, indicating that oil could directly penetrate through the S-PLA-FSPS surface. The above analysis can be explained that the selective and rapid oil–water separation can be achieved by the S-PLA-FSPS membrane with appropriate driving force.

### 2.4. Adsorption Performance

**Figure 8:**
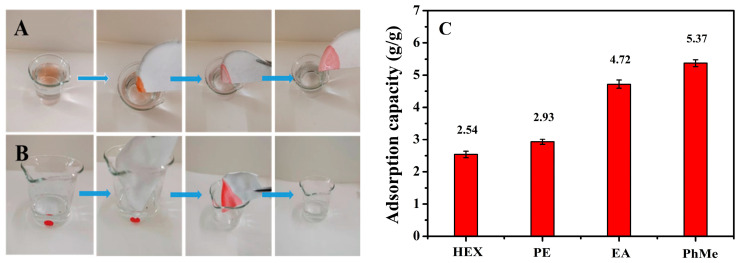
Adsorption experiments of light oil (**A**), heavy oil (**B**), adsorption capacity (**C**).

The surface area and pore volume of the membranes were S-PLA-FSPS 43 m^2^/g and 0.15 cm^3^/g, respectively, by nitrogen adsorption and desorption experiments at room temperature (Figure S10). Due to the rich porous structure of the S-PLA-FSPS membrane, it had a certain adsorption effect on oil. Therefore, it could also be applied to the adsorption and removal of floating oil on water and heavy oil underwater. As shown in Figure 8, the membrane showed the excellent adsorption effect on both light oil (Figure 8A and Video S2) and heavy oil (Figure 8B and Video S3). Adsorption experiments of n-hexane, petroleum ether, ethyl acetate, and methylbenzene were performed within 5 min with S-PLA-FSPS membranes with a thickness of only 0.55 ± 0.05 mm (Figure 8C), the results show that the adsorption capacities for these organic reagents were 2.54 ± 0.55, 2.93 ± 0.21, 4.72 ± 0.28, and 5.37 ± 0.14 g/g, respectively. Therefore, the PLA membrane with the excellent adsorption capacity also had the application value in the field of oil removal and water purification.

## 3. Experimental

### 3.1. Materials

PLA was provided by Shanghai McLean Biochemical Co., Ltd., Shanghai, China (PLA, Mw: 110,000, particle size: 3 mm), 1,4-dioxane (DiOX, AR, 99.5%) was provided by Sinopharm Chemical Reagent Co., Ltd., Shanghai, China. The reagents and medicines provided by Xilong Science Co., Ltd., Shantou, China. was dichloromethane (DCM, AR), acetic acid (AR), methylbenzene (PhMe, AR), methylene blue (AR). Dimethyl sulfoxide (DMSO, AR) was provided by Tianjin Damao Chemical Reagent Factory, Tianjin, China. N-hexane (HEX, AR), petroleum ether (PE, AR), and ethyl acetate (EA, AR) were provided by Guangdong Guanghua Technology Co., Ltd., Shantou, China, and Sudan Red III (AR) was provided by Shanghai Ron Reagent, Shanghai, China.

### 3.2. Preparation of PLA Membranes

The PLA membranes were prepared by the freeze solidification phase separation method (PLA-FSPS). To be specific, 0.5 g of PLA was added into 10 mL of DCM and DiOX mixed solvent and sufficiently dissolved, then the mixture was heated with magnetic stirring at 30 °C for 3 h to with a uniform concentration of 50 mg/mL. The obtained solution was poured into a glass petri dish, frozen at −7 °C for 3 h, then taken out, and left to dry naturally at room temperature for 24 h. The effect of different solvent ratios (DiOX:DCM to 10:0, 9:1, 4:1, 1:1, and 0:10) on membrane structure was investigated. In order to further improve the superhydrophobicity of PLA, the semi-cured PLA membrane was impregnated in a mixed solution after the frozen PLA was placed at room temperature for 6 h. The solution impregnation was composed of deionized water and acetic acid with different volume ratios (1:0, 1:1, and 0:1). The PLA-FSPS membrane after solution impregnation for 2 h was washed with deionized water, and then placed in an oven at 60 °C to dry, and the PLA functional membrane (S-PLA-FSPS) was finally obtained. By comparison, the PLA membranes were prepared by the solution casting method (PLA-SC): The solution with a uniform concentration of 50 mg/mL obtained above was poured into a glass petri dish and air-dried naturally. 

### 3.3. Oil–Water Separation Experiments

**Figure 9:**
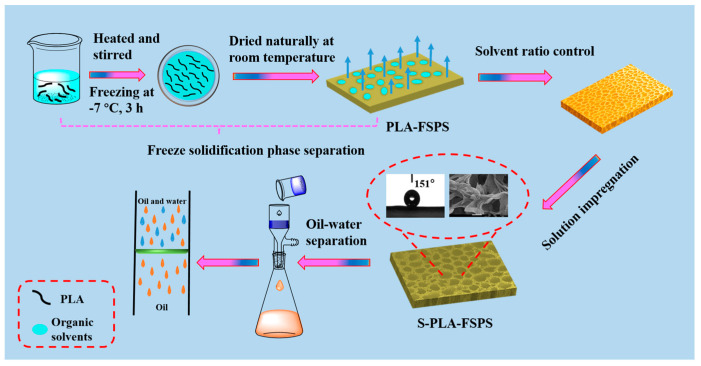
Schematic diagram of preparation of S-PLA-FSPS membrane and oil–water separation.

The schematic diagram of the preparation of PLA membrane and the experimental schematic of oil–water separation is shown in Figure 9. The deionized water and n-hexane organic reagent mixed in a volume ratio of 1:1. For the better visual distinction, the deionized water was stained with methylene blue dye, and n-hexane organic reagent was stained with Sudan red III. The prepared S-PLA-FSPS samples were fixed in a filter device with an inner diameter of 4 cm. The oil–water mixture was poured into the filter device, and filtered under only gravity. In order to examine the reusability of the membrane, 20 cycle tests were carried out. The separation efficiency R (%) was calculated as follows:

R = (V_2_/V_1_) × 100% (2)

Among them, V_1_ (L) and V_2_ (L) are the volumes before and after separation, respectively.

The calculation formula of membrane flux J (L·m^−2^·h^−1^) was as follows:

J = V/(A × △t)(3)

V (L) represents the volume of the filtered solution, A (m^2^) represents the effective area of the membrane, and t (h) represents the separation time.

### 3.4. Oil Adsorption Experiment

The adsorption experiments of n-hexane, petroleum ether, ethyl acetate, and toluene were carried out using the sample membrane, and the calculation formula of the adsorption capacity was as follows:

Q = (m_1_ − m_0_/m_0_) × 100% (4)

where, m_0_ and m_1_ represent the mass before and after adsorption, respectively.

### 3.5. Characterization

The surface morphology of samples was tested by athermal field emission scanning electron microscope (SEM, JEOL Ltd., Akishima, Tokyo, JSM-7100F), and the samples were sprayed with gold before the test. The vacuum degree was 5 × 10^−4^ Pa, and the voltage was 5 KV. The surface wettability of samples was tested by a contact angle goniometer (CA, OCA15EC, Dataphysics, Filderstadt, Germany), using the dropping method at room temperature, and 2 μL of deionized water and n-hexane reagents were slowly dropped on the surface of the PLA samples. Each sample was repeated five times and averaged. The Fourier transform infrared spectrometer (FT-IR, Thermo Scientific Nicolet 6700, Waltham, MA, USA) was used to determine the structure of samples. The crystal structure of samples was determined by the X-ray diffractometer (XRD, Rigaku SmartLab 9 Kw, Rigaku Corporation, Japan) with the 2θ range of 5~40° at a scan speed of 5°/min. The pore size and pore size distribution of samples were determined by the capillary flow pore size analyzer (Porolux100, Porometer Ltd., The Woodlands, TX, USA), the samples were cut to 25 mm gauge, and the dry and wet samples were tested under 2~6 bar pressure. The crystallization behavior of samples was tested by the differential scanning calorimeter (DSC, DSC25, TA Instruments, New Castle, DE, USA) under nitrogen atmosphere at the initial equilibrium temperature of −50 °C, and the temperature increased to 200 °C at a rate of 10 °C/min to measure the DSC heating curve. The thermal stability of the membrane was tested by a METTLER TOLEDO thermal analyzer (TGA, Netzsch Sta 2500, Mettler Toledo, Switzerland). The pore structure properties of the membranes were measured by Autosorb-iQ (BET, Quantachrome, Konta, CA, USA) nitrogen adsorption apparatus. The mechanical properties of the membranes (5 mm × 50 mm) were measured by electronic universal tensile testing machine (WDW-1, China) at a speed of 50 mm/min. The surface roughness of the samples was tested by the atomic mechanical microscopy (AFM, Bruker Dimension Icon, Bruker, Germany). The reflectance spectra of membranes were measured in the range of 200–800 nm by using a UV-vis diffuse reflectometer (Shimadzu UV-2600, Shimadzu, Tokyo, Japan). The porosity of the membrane was calculated by the gravimetry of the liquid contained in the pores of the membrane. The membrane samples were immersed in isobutanol for 12 h. The formula for calculating the porosity of the membrane was as follows:

ε = (W_w_ − W_d_/(A × d × ρ)) ×100% (5)

where W_w_ is the weight of the wet membrane (g), W_d_ is the weight of the dry membrane (g), A is the membrane area (cm^2^), d is the membrane thickness (cm), and ρ is the density of isobutanol (0.8 g/cm^3^).

## 4. Conclusions

The environmentally friendly and superhydrophobic PLA membrane with tunable pore size was successfully prepared by a simple and efficient freeze solidification phase separation method. Compared with the solution casting method, freeze solidification phase separation method used frozen solvent and thawed the solvent at room temperature to obtain porous PLA membranes. By adjusting the pore size and structure of the membrane by different solvent ratios and solution impregnation induction, the porous S-PLA-FSPS membrane with a micro-nano-scale pleated structure was obtained. The surface roughness Ra was 338 nm, and the contact angle to water was 151°. The membrane flux used for oil–water separation reached 16,084 L·m^−2^·h^−1^, and the water separation efficiency was 99.7%, which were much higher than that of other oil–water separation materials. In addition, the S-PLA-FSPS membrane could also be applied for the adsorption and removal of oil slick and underwater heavy oil and provide ideas for solving the problems of water and oil pollution and oil leakage.

## Figures and Tables

**Table 1:** The pore size parameters of PLA-FSPS membranes with different solvent ratios.

DiOX:DCM	10:0	9:1	4:1	1:1	0:10
Maximum aperture/μm	0.46	6.10	6.10	2.29	--
Average aperture/μm	0.54	0.98	1.32	0.35	--
Minimum aperture/μm	0.25	0.68	0.51	0.29	--

**Table 2:** Comparison with similar work in recent years.

Material	Drive	Membrane Flux	Separation Efficiency	Reference
TiO_2_-PLA	1 bar	963 L·m^−2^·h^−1^	99%	[5]
PDMS/SNPs-PI	Gravity	4400 L·m^−2^·h^−1^	99.55%	[7]
Grphene/Poly(vinyl alcohol) janus aerogels	Gravity	1306 L·m^−2^·h^−1^	99.7%	[47]
Fe^3+^-PA/OTMS/PI	Gravity	8424 L·m^−2^·h^−1^	99%	[48]
PVDF-g-SiO_2_NPs/PAMAM membrane	0.9 bar	>3100 L·m^−2^·h^−1^	>99%	[49]
MCNF-membrane	Gravity	3730 L·m^−2^·h^−1^	99%	[50]
Polybenzoxazine-coated cotton fabric	Gravity	7200 L·m^−2^·h^−1^	99%	[51]
Cellulose-starch silica composite coating nylon membrane	1 bar	31,847 L·m^−2^·h^−1^	99.8%	[52]
GO and rGO coated cotton fabric	Gravity	7120 L·m^−2^·h^−1^	98.5%	[53]
S-PLA-FSPS membrane	Gravity	16,084 L·m^−2^·h^−1^	99.7%	This work

## Data Availability

Not applicable.

## Supplementary Materials

The following supporting information can be downloaded at: https://www.mdpi.com/article/10.3390/molecules28145590/s1, Figure S1. Reflectance test of PLA-SC and PLA-FSPS. Figure S2. Images of PLA-FSPS membrane surface (A) and cross-section (B). Figure S3. TG and DSC curves of PLA-FSPS membrane. Figure S4. The maximum stress and strain of PLA-FSPS membrane. Figure S5. The oil-water separation test of PLA-SC and PLA-FSPS. Figure S6. CA test images of PLA-FSPS membranes. Figure S7. Pore size distribution of the membranes prepared by different ratios of solvent. Figure S8. The oil-water separation test. Figure S9. Superoleophilicity of membrane. Figure S10. Nitrogen adsorption-desorption isotherms of membrane. Table S1. Membrane aperture parameters of the membranes prepared by different methods Table S2. Membrane aperture parameters of the membranes with solution impregnation induced.
