# Supplementary material for: The Preparation of Superhydrophobic Polylactic Acid Membrane with Adjustable Pore Size by Freeze Solidification Phase Separation Method for Oil–Water Separation
Source: Molecules. 2023 Jul 22;28(14):5590. doi: 10.3390/molecules28145590 (PMC10384457; doi:10.3390/molecules28145590)
Supplement: Supplementary file 1 [file molecules-28-05590-s001.zip › Supporting Information.pdf]

Supporting Information

The preparation of superhydrophobic polylactic acid membrane with adjustable pore size by freeze solidification phase separation method for oil-water separation

Yan Zhang[a,b], Tianyi Sun[a,b*], Dashuai Zhang[a,b], Shishu Sun[a,b], Jinrui Liu[a,b], Bangsen Li[a,b] and Zaifeng Shi[a,b*]

[a]Key Laboratory of Water Pollution Treatment & Resource Reuse, Hainan Normal University, Haikou, Hainan 571158, P.R. China

[b]College of Chemistry and Chemical Engineering, Hainan Normal University, Haikou, Hainan 571158, P.R. China

* Corresponding author, Dr. Tianyi Sun e-mail: tianyi870328@163.com, Professor Dr.Zaifeng Shi e-mail: zaifengshi@163.com,

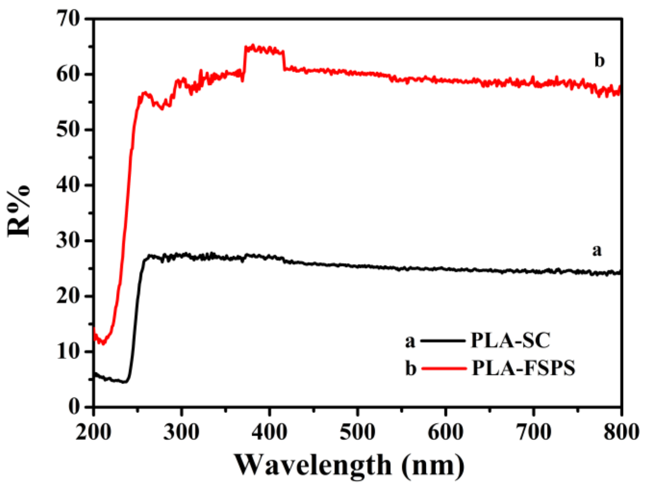

Fig.S 1 Reflectance test of PLA-SC and PLA-FSPS.

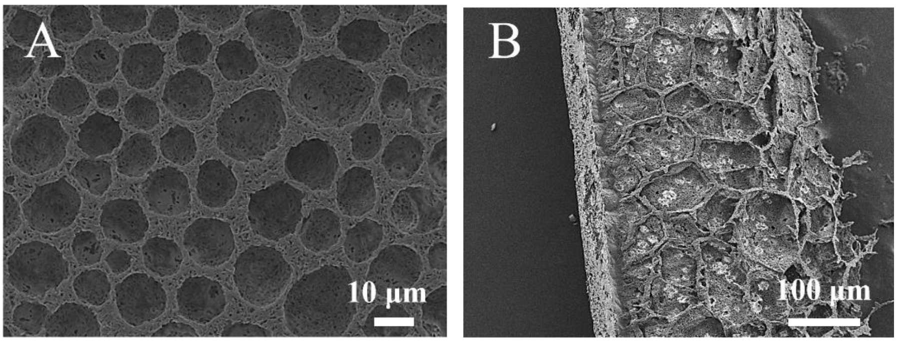

Fig.S 2 Images of PLA-FSPS membrane surface (A) and cross-section (B).

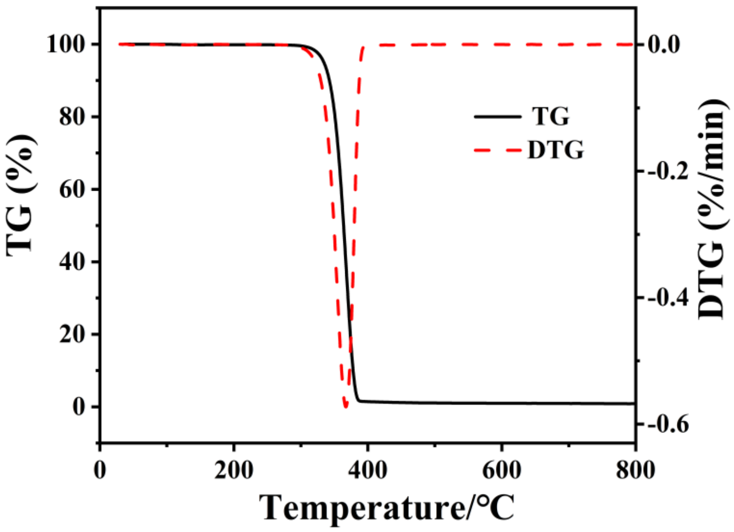

Fig. S 3 TG and DSC curves of PLA-FSPS membrane.

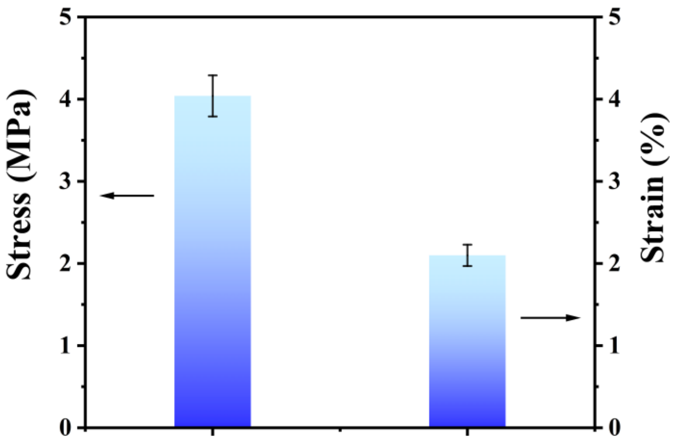

Fig. S 4 The maximum stress and strain of PLA-FSPS membrane.

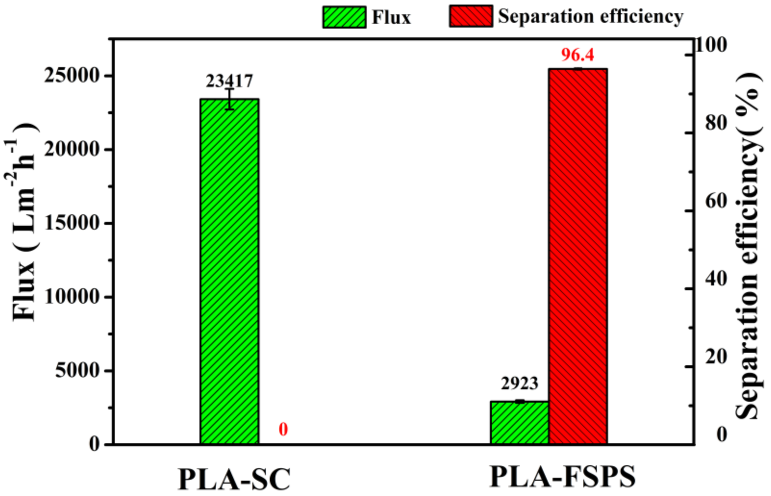

Fig.S 5 The oil-water separation test of PLA-SC and PLA-FSPS.

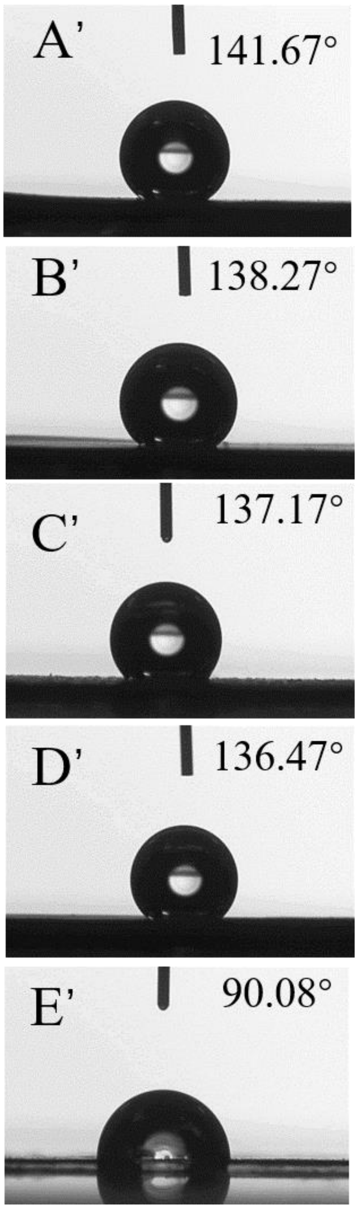

Fig.S 6 CA test images of PLA-FSPS membranes.

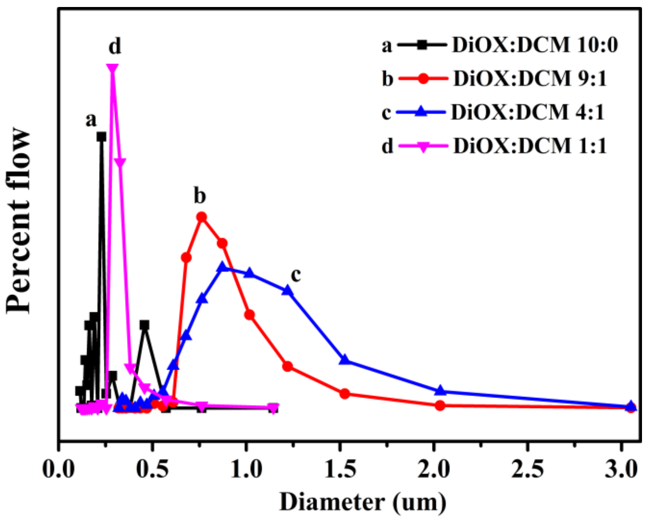

Fig. S 7 Pore size distribution of the membranes prepared by different ratios of solvent.

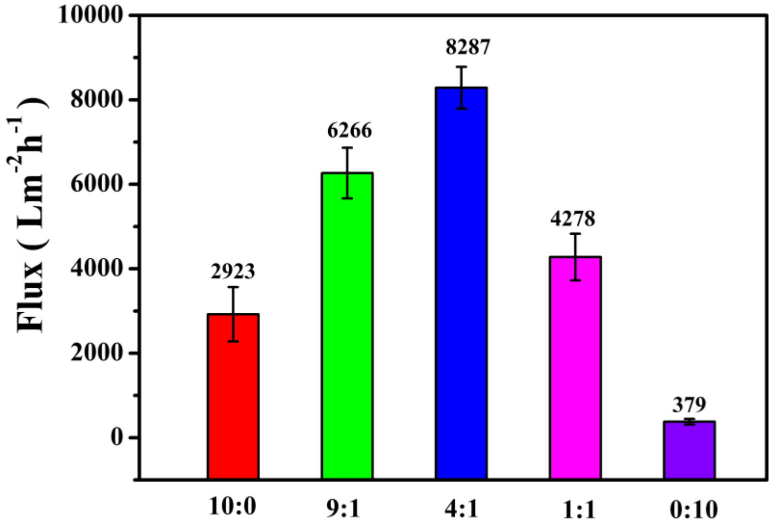

Fig. S 8 The oil-water separation test.

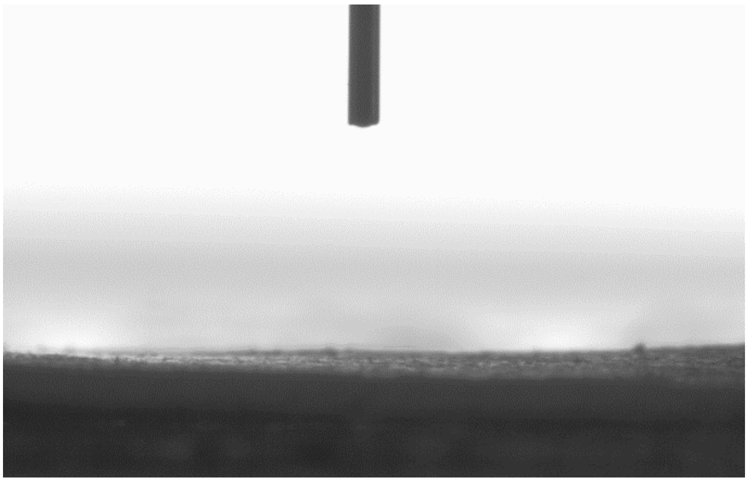

Fig. S 9 Superoleophilicity of membrane.

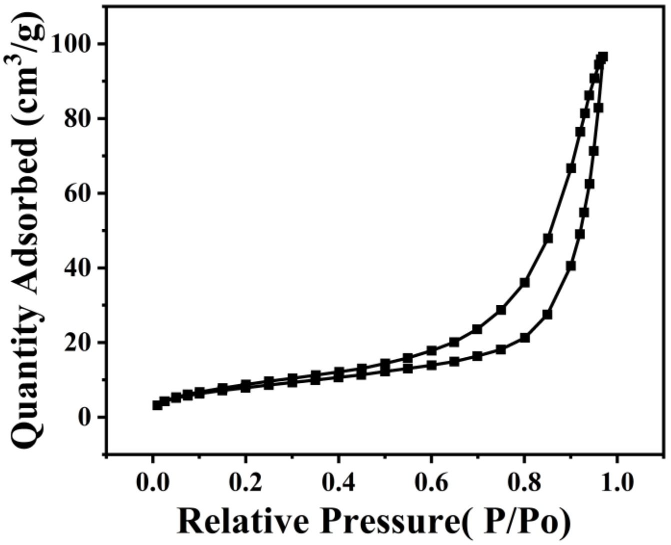

Fig. S 10 Nitrogen adsorption-desorption isotherms of membrane.

Table S 1 Membrane aperture parameters of the membranes prepared by different methods

Specification	Solution casting method	Freeze-solidification phase separation method
Maximum aperture/μm	71.30	0.54
Average aperture/μm	25.86	0.46
Minimum aperture/μm	2.35	0.25
Porosity/%	51.01±0.07	77.60±0.06

Table S 2 Membrane aperture parameters of the membranes with solution impregnation induced

Specification	No solution impregnation induction	Deionized water	Acetic acid	Deionized water and acetic acid 1:1
**Maximum aperture/μm**	**8.96**	**11.4**	**11.13**	**11.14**
**Average aperture/μm**	**1.15**	**1.25**	**3.91**	**4.05**
**Minimum aperture/μm**	**0.48**	**0.74**	**1.00**	**0.99**
